# Generation of fluorescently labeled tracers – which features influence the translational potential?

**DOI:** 10.1186/s41181-017-0034-8

**Published:** 2017-12-15

**Authors:** Fijs W. B. van Leeuwen, Bart Cornelissen, Federico Caobelli, Laura Evangelista, Latifa Rbah-Vidal, Silvana Del Vecchio, Catarina Xavier, Jacques Barbet, Marion de Jong

**Affiliations:** 1 Interventional Molecular Imaging Laboratory, Department of Radiology, Leiden University Medical Center, Leiden, the Netherlands; 2 Department of Oncology, CRUK&MRC Oxford Institute for Radiation Oncology, University of Oxford, Oxford, UK; 3 Department of Nuclear Medicine, University Hospital Basel, Basel, Switzerland; 4 Nuclear Medicine and Molecular Imaging Unit, Veneto Institute of Oncology IOV – IRCCS, Padua, Italy; 5 CRCINA, INSERM, CNRS, Université d’Angers, Université de Nantes, Nantes, France; 6 Department of Advanced Biomedical Sciences, University Federico II, Naples, Italy; 7 In vivo Cellular and Molecular Imaging Lab (ICMI)-Department, Vrije Universiteit Brussel, Ixelles, Belgium; 8 Department of Radiology & Nuclear Medicine, Erasmus Medical Centre, Rotterdam, the Netherlands

**Keywords:** Fluorescence, Image guided surgery, Molecular imaging, Tracers, Nuclear medicine, Dual-modality

## Abstract

Given the increasing exploration of fluorescent tracers in the field of nuclear medicine, a need has risen for practical development guidelines that can help improve the translation aspects of fluorescent tracers. This editorial discusses the does and don’ts in developing fluorescence tracers. It has been put forward by the European Association of Nuclear Medicine (EANM) Translational Molecular Imaging & Therapy committee and has been approved by the EANM board.

## Introduction

With the upcoming field of image-guided surgery, not only traditional ^99m^Tc- and ^111^In-labelled radioguidance procedures blossom, but also the development of fluorescent and hybrid/bimodal tracers gains increasing interest. While converting efficient ^68^Ga-PET tracers for e.g. Prostate Specific Membrane Antigen to ^99m^Tc- labeled versions for radioguided surgery (Robu et al., 2017) is already part of the traditional radiochemistry skillset, attachment of fluorescent dyes as imaging labels requires additional expertise. Surprisingly, it is not fully recognized yet which modifications can be induced by the addition of fluorescent dyes, e.g. alterations in biodistribution, and manuscripts seldomly report the key analytical features required to objectively analyze them. In this editorial we aim to discuss a number of factors to be taken into consideration when fluorescent dyes are used in in vivo imaging tracers.

### Spectral properties

Fluorescence, a type of luminescence, is the result of the excitation of a conjugated system following the absorbance of light. When the excited electron reverts back to its ground state it generates light with a longer wavelength (emission). In fluorescent organic dyes, electrons that can absorb high-energy photons are delocalized in connected p-orbitals due to alternating single and double bonds (e.g. =C-C = C-). The absorbance spectrum (nm) and absorption maximum of dyes, and thus the in-depth excitability during an in vivo application (van Leeuwen et al., 2015), depends on the size and composition of the dye: the bigger the conjugated system, the longer the wavelength it can absorb. This feature is well illustrated by cyanine dyes Cy3, Cy5, and Cy7, which only differ in their bridge length, but display an absorption maximum shift ranging from around 550 nm (visible) over 650 nm (far-red) to around 750 nm (near-infrared). Simply this means that a quest for near-infrared dyes converts to the use of large fluorescent labels and with that increases the risk that the fluorescent label negatively influences the tracer pharmacokinetics. The efficiency of a molecule absorbing light, the molar extinction coefficient (ε), also depends on the composition and surroundings of the dye, as does the conversion efficacy of the photons absorbed into emitted photons, the quantum yield (φ).

Since the brightness of a fluorescent dye is proportional to the product of ε and φ, it is compulsory that these should be accurately recorded and reported for each novel dye. It should be noted that these signature parameters could be considered as specific and critical as the emission types and lifetimes of the radionuclides commonly applied in nuclear medicine. Contrary to radionuclides, the environment wherein the dyes reside strongly influences their photophysical properties. Thus, researchers should realize that these properties might vary when the dyes are conjugated to different vectors (e.g. peptide vs. protein) or when different pH values or solvents are used for analysis (e.g. serum vs. dimethylsufoxide (DMSO)). Hence, these features should ideally be recorded for each individual tracer and should be measured in formulations that are representative for in vivo applications. For example, measuring the φ in DMSO could overestimate its value in an other environment). This is exemplified by indocyanine green, which has a φ of 12% in DMSO, yet 2% in saline buffer (Benson & Kues, 1978).

**Fig. 1:**
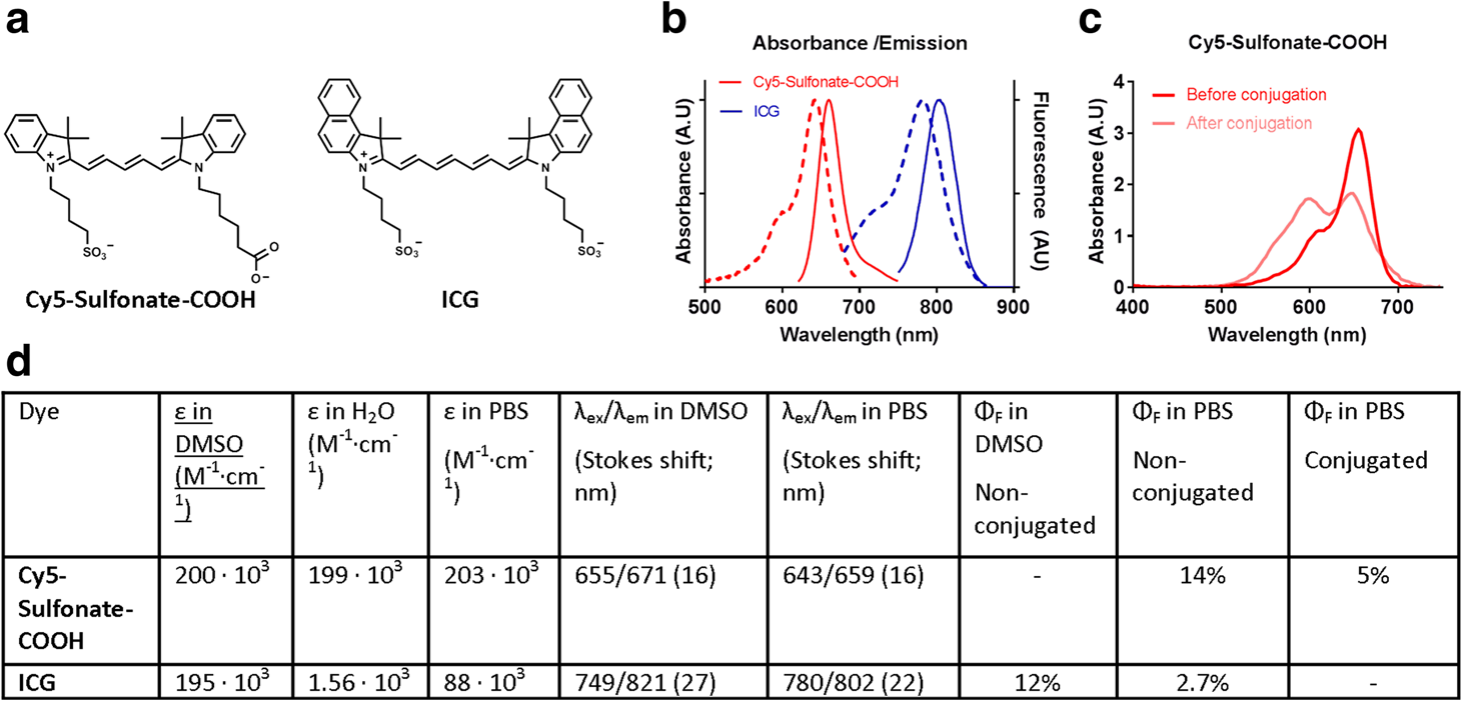
Key features in the analysis of fluorescence tracers illustrated using (**a**) the conjugatable cyanine dye Cy5-sulfonate-COOH and the widely clinically applied dye Indocyanine green (ICG), **b** Absorbance and emission spectrum of the non-conjugated dyes Cy5-sulfonate-COOH and ICG, **c** Changes in absorption spectrum as a result of conjugation, **d** Relevant photophysical parameters and their solvent dependence

Unfortunately, for vectors with multiple conjugation sites, the quenching interactions between the dyes present on the same vector can influence the brightness of the tracer. Aggregating dyes residing on the same vector may quench each other’s fluorescence, a feature most pronounced for slightly lipophilic and symmetrical dyes (van der Wal et al., 2016). It has even been shown that this effect can also occur between different types of dyes (Rood et al., 2014). Dyes that reside in 8–10 nm vicinity of each other may display Förster resonance energy transfer, again limiting their fluorescence intensity. Therefore, labeling ratios should be determined utilizing the Beer-Lambert law. Such analyses may, however, still be limited by the fact that stacking effects, either the result of non-covalent or covalent interactions, alter the absorbance profile of dyes. Stacking typically yields an additional lower absorbance peak in the case of cyanine dyes (see Fig. 1). Hence, for an objective assessment of the number of dyes on e.g. a monoclonal antibody (mAb) it is key that authors, in addition to the above features, provide an absorbance spectrum of their compound in the formulation that is applied to patients.

### Chemical radio- and photo-stability

When administering tracers in vivo, stability becomes an issue, as metabolites may illuminate non-specific features. Enzymatic degradation of peptides is common (Nock et al., 2014), but peptidases are limited in their ability to metabolize synthetic dyes. Nevertheless, removal of the dye form the targeting vector should be deeply investigated. Degradation of the dye is most probable as the result of a nucleophilic attack on aryl ethers by primary amines or thiols, which often occurs in an in vivo environment. Previously reported studies on these interactions indicate that they may induce dye disintegration and a reduction of fluorescence intensity of e.g. ZW800-I (Hyun et al., 2014). Alternatively, thiol-based reactions with NIRDye 800 CW under the same conditions allow formation of entirely new molecules (van der Wal et al., 2016). Both features are highly undesirable and should be evaluated and documented prior to using vectors labeled with dyes in vivo, particularly in humans.

As the intensity of the light emission is directly related to the absorption of light by a fluorescent dye, manufacturers commonly enhance system performance by increasing the intensity of the excitation light source. Although this theoretically represents a valid approach, such modifications can also result in disintegration of the dye. This effect (photo-bleaching) is well known in molecular cell biology (Hoebe et al., 2007). As the bleaching behavior is highly dye- and camera-dependent, its occurrence might prevent the surgical identification of lesions under fluorescence guidance. Hence, the evaluation of photo-stability of a fluorescent tracer in combination with the fluorescence camera intended for in vivo use is a key aspect in the translational process (van der Wal et al., 2016).

A recent topic of interest has been the radio-stability of dyes in hybrid/bimodal tracers. It is not surprising that if dyes can be photo-bleached, irradiation, in particular β- or α-emission, can induce radio-bleaching (Hernandez et al., 2017). This effect, which is radiation dose-dependent, is most likely to occur as result of prolonged exposures in the reaction mixture following radiolabeling, and does not occur when solutions of bimodal or hybrid tracers are used directly after production. To extend the shelf life of the reaction mixture, electron-scavengers can be applied to the formulation. As in vivo tumor accumulation commonly occurs at a relatively low concentration (< 10% ID/g), radiobleaching is not likely to influence the in vivo use of such tracers.

### Pharmacokinetics and quantification

Given that the fluorescent dyes used for in vivo imaging are relatively large molecules, it should come as no surprise that their conjugation may severely affect the affinity and in vivo kinetics of any targeting vector. Not only could the dye negatively influence receptor interactions by inducing non-specific binding (Santini et al., 2016), it may also influence the cellular localization of some smaller vectors (Berkers et al., 2007). While such influences were expected for relatively small peptide-based vectors (Bunschoten et al., 2016), they have also been reported for much larger compounds, such as mAbs (Cohen et al., 2011; Zhou et al., 2014). One key parameter in the in vivo pharmacokinetics is the dye’s interaction with serum proteins such as albumin. For ICG for example, this effect is so strong that it allows formation of non-covalent ICG-Albumin complexes (Bunschoten et al., 2012). Hence, it seems desirable to quantitatively document these interactions for new tracers.

Fluorescence detection alone allows quantitative in vitro analysis, but quantitation of in vivo effects are most practical for tracers that include a radiolabel. While critical for the assessment of toxicity-related aspects, this information is generally missing in clinical reports on the use of fluorescent tracers (van Dam et al., 2011; Rosenthal et al., 2015; Burggraaf et al., 2015; Lamberts et al., 2017). Given the proven and well-accepted potential of radionuclide-based assessments by pharma and academia during drug and tracer development, it seems reasonable to advocate a quantitative pharmacokinetic assessment to become a standard requirement in reports on novel fluorescent tracers.

### Injected dose and cost impact

Brightness, optical and in vivo stability, as well as pharmacokinetic properties combined dictate the tracer amount required for in vivo fluorescence imaging. In general, clinical trials use milligrams of fluorescent compounds, making them financially less achievable for most hospitals. At the same time high doses may potentially lead to (partial) saturation of low capacity targets. Trials with hybrid tracers indicate that lower injected doses would still allow for fluorescence-based lesion identification (KleinJan et al., 2016). Importantly, the amount of tracer required for efficient imaging is directly reflected in the cost of toxicity assessments and ultimately the cost of the tracer. In addition, the potential cost reduction to the overall healthcare system is critical for reimbursement and wider clinical acceptance, and health economical assessments are an extremely useful inclusion when clinical trials with novel imaging agents are proposed. A relatively simple estimation of potential reimbursement cost for effective intraoperative visualization is the (% occurrence of failure) x (the average costs of follow up e.g. repeat surgery). For example, in expert surgical centers, breast cancer surgery is only irradical in 5% of the cases. Assuming that the cost of a repeat surgery is €5000 euro, reimbursement of a tracer that allows for a theoretical 100% radical resection rate will theoretically come to a value of €250 euro. Obviously this number is higher for surgical procedures with a higher failure rate and may increase if a clear improvement in the patient’s quality of life can be realized. Nevertheless, cost efficiency should always be taken into account when there are true translational aspirations for (fluorescent) tracers.

## Conclusions

Future translation of fluorescent imaging agents strongly relies on more thorough documentation of the compound’s photo-physical properties, chemo-, photo- and radio-stability, as well as translational aspects such as pharmacokinetics, dose and cost.
